# ‘Double-muscling’ and pelvic tilt phenomena in rabbits with the cystine-knot motif deficiency of myostatin on exon 3

**DOI:** 10.1042/BSR20190207

**Published:** 2019-05-21

**Authors:** Ting Zhang, Yaoyao Lu, Shaozheng Song, Rui Lu, Minya Zhou, Zhengyi He, Tingting Yuan, Kunning Yan, Yong Cheng

**Affiliations:** 1 College of Veterinary Medicine, Yangzhou University, Yangzhou 225009, Jiangsu, China; 2 Jiangsu Co-innovation Center for Prevention and Control of Important Animal Infectious Diseases and Zoonoses, Yangzhou 225009, Jiangsu, China; 3 Institute of Translational Medicine, Medical College, Yangzhou University, Yangzhou 225009, Jiangsu, China; 4 School of Nursing, Taihu University of Wuxi, Wuxi 214000, Jiangsu, China

**Keywords:** cystine-knot structure, CRISPR/Cas9, double-muscling, MSTN, pelvic tilt, rabbits

## Abstract

Gene mutations at different gene sites will produce totally different phenotypes or biological functions in gene-edited animals. An allelic series of mutations in the myostatin (*MSTN*) gene can cause the ‘double-muscling’ phenotype. Although there have been many studies performed on *MSTN*-mutant animals, there have been few studies that have investigated the cystine-knot motif in exon 3 of *MSTN* in rabbits. In the current study, CRISPR/Cas9 sgRNA anchored exon 3 of a rabbit’s *MSTN* was used to disrupt the cystine-knot motif to change the *MSTN* construction and cause a loss of its function. Eleven *MSTN*-KO founder rabbits were generated, and all of them contained biallelic modifications. Various mutational MSTN amino acid sequences of the 11 founder rabbits were modeled to the tertiary structure using the SWISS-MODEL, and the results showed that the structure of the cystine-knot motif of each protein in the founder rabbits differed from the wild-type (WT). The *MSTN*-KO rabbits displayed an obvious ‘double-muscling’ phenomena, with a 20−30% increase in body weight compared with WT rabbits. In the *MSTN*-KO rabbits, all of the *MSTN^−/−^* rabbits showed teeth dislocation and tongue enlargement, and the percentage of rabbits having pelvic tilt was 0% in *MSTN*^+/+^, 0% in *MSTN*^+/−^, 77.78% in female *MSTN*^−/−^ rabbits, and 37.50% in male *MSTN*^−/−^ rabbits. The biomechanical mechanism of pelvic tilt and teeth dislocation in the *MSTN*-KO rabbits requires further investigation.

These newly generated *MSTN*-KO rabbits will serve as an important animal model, not only for studying skeletal muscle development, but also for biomedical studies in pelvic tilt correction and craniofacial research.

## Introduction

Myostatin (MSTN) can negatively regulate the growth and development of skeletal muscle. It is a member of the transforming growth factor β (TGF-β) superfamily of growth and differentiation factors [1,2]. MSTN functions to inhibit excessive growth of muscle in order to maintain an overall balance of this tissue relative to adipose tissue [3]. Natural *myostatin* null animals, including cattle [4], dogs [5], humans [6], and sheep [7], shows a dramatic and widespread increase in skeletal muscle mass; namely, a ‘double-muscling’ phenomenon.

Since its discovery in mice [8], *MSTN* has been extensively investigated in other animals. A large number of variants have been identified in cattle, most of which are silent or neutral. Two of the variants in the third exon strongly affect the ‘double-muscling’ phenotype in Belgian Blue cattle [9]. An 11-bp deletion at the bovine *myostatin* nucleotide 821 [nt821(del11)] in the coding region results in a frameshift mutation. Also, in Piedmontese cattle [4], a G to A transition at nucleotide 938 results in the substitution of cysteine by tyrosine (this mutation is referred to as C313Y). It has been shown that an 11-bp deletion [nt821(del11)] in the *myostatin* gene results in a truncation of the bioactive carboxyterminal domain of the protein [9,10]. In addition, the C313Y mutation predicts the replacement of cysteine at amino acid 313 with tyrosine. This cysteine is the fifth in a series of nine whose appearance and spacing is extremely conserved among TGF-β family members [2,4]. These two mutations remove the portion of the myostatin protein that is most highly conserved among TGF-β family members. The rabbit *myostatin* gene consists of three exons and two introns. The third exon of the rabbit *myostatin* gene codes for mature processed myostatin, which is the active region of the molecule and shares extensive homology with other TGF-β members [2,11]. Similar to other TGF-β family members, three kinds of proteins are translated by *myostatin*: a precursor protein consisting of an N-terminal signal sequence; a propeptide domain; and a growth factor domain that contains the characteristic cystine-knot motif (C272–C282, C281–C340, C309–C372, and C313–C374). C339 forms the intermolecular dimerization disulfide bond [12,13]. The C313Y mutation removes the C313-containing disulfide bond, an integral part of the characteristic TGF-β cystine-knot structural motif. Some research has suggested that the cystine-knot motif, which is essential for myostatin structural stability and covalent dimerization, is required for receptor-mediated signaling by myostatin growth factor *in vivo*, with its absence leading to a double-muscled phenotype (‘double-muscling’) [14]. Hence, it is feasible to prepare a rabbit model of myostatin using the knockout cystine-knot motif.

Previous studies have reported that *MSTN* naturally mutant cattle generally show a 20−40% increase in muscle weights due to muscle hypertrophy or hyperplasia relative to wild-type (WT) cattle [15]. This is consistent with a report that found that individual muscle fibers of homozygous *MSTN*-KO mice (*MSTN*^−/−^ mice) produced by the homozygous deletion of the C-terminal region of the *myostatin* gene in embryonic stem cells were double or triple in mass compared with those of their heterozygous and WT littermates [2]. Some research has reported that the masseter and temporalis muscles of *MSTN^−/−^* mice were over 50% larger in mass than WT controls due to larger cross-sections of the muscle fibers and increased numbers of muscle cells [2,16]. Therefore, *MSTN^−/−^* mice were used to explore the effects of increased muscle mass on sagittal suture complexity. The mice were also used to indicate that a consistent amount of force needs to be applied over a much greater area of the craniofacial skeleton in the *myostatin* knockout, and this should, in turn, lead to altered biomechanical stress and bony morphology [3,16]. An *MSTN^−/−^* mouse has significantly larger temporalis, masseter, and medial and lateral pterygoid muscles than WT controls [17]. These characteristics, coupled with morphologic findings of the skull in *MSTN^−/−^* mice (e.g., shorter crania, longer, and rounder mandibles), provide further evidence of an altered craniofacial loading environment due to *MSTN* deficiency [3,16–20]. Therefore, these *MSTN*-KO mice have been used as a model for studying muscle–bone interactions, such as to investigate the role of increased mandibular elevators and bite forces on temporomandibular joint morphology. In the past 5 years, many *MSTN*-KO animals (e.g., pigs, goats, sheep, and rabbits) with ‘double-muscling’ have been generated due to the emergence of the CRISPR/Cas9 gene editing technique as a newly versatile genome engineering tool [21–24]. However, the morphology and the muscle–bone interactions of these *MSTN*-KO animals have rarely been investigated.

The aim of the present study is to generate *MSTN*-KO rabbits using the CRISPR/Cas9 system anchored to the cystine-knot motif by microinjecting Cas9 mRNA and sgRNA into fertilized rabbit eggs. The *MSTN*-KO rabbits can be used as a basis to create new breeds of rabbit to improve muscle yield, with the ultimate goal of approval for human consumption. In addition, an important animal model is proposed for use in further studies that investigate muscle–bone interactions.

## Results

### CRISPR/Cas9 expression plasmid construction and *in vitro* transcription

**Figure 1:**
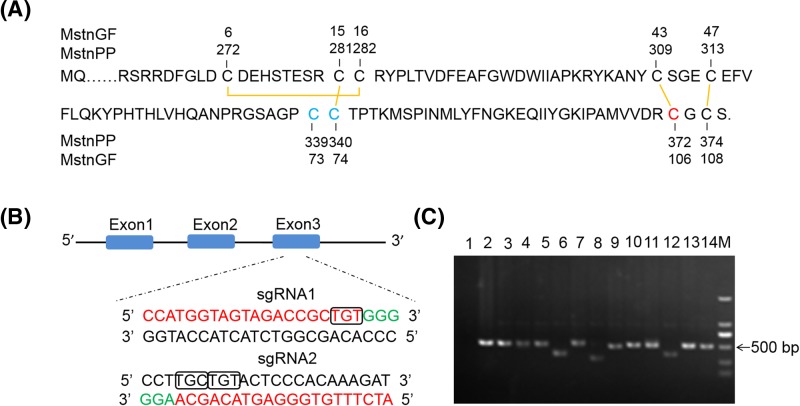
Schematic diagram of sgRNA targeting the rabbit *MSTN* gene loci (**A**) The myostatin growth factor primary structure. Disulfide bonds are shown in yellow. C339 and C340 highlighted in blue are targeted by sgRNA-2, and C372 in red is targeted by sgRNA-1. Sequence numbering for the full-length precursor myostatin is shown. (**B**) Two sgRNA sequences, sgRNA-1, and sgRNA-2 are marked in red, and the protospacer adjacent motif (PAM) sequences are presented in green. TGT with a black box encodes C372 in the sgRNA-1 sequence and TGC and TGT with black boxes encode C339 and C340 separately. (**C**) The PCR products of electrophoresis of the rabbit *MSTN* gene. 1: Double-distilled water as a blank control; 2: The DNA of a WT rabbit as a negative control; 3−9: M1−M7; 10−14: M8−M12; M: DL2000 DNA marker.

To produce gene-modified New Zealand white rabbits with double-muscling, two sites that are associated with the cystine-knot motif in the *MSTN* rabbit gene were selected as target genes (Figure 1A,B). The sgRNA-1 anchors the *MSTN* gene site that encodes C372, and the sgRNA-2 anchors the *MSTN* gene site that encodes C339 and C340.

### Generation of *MSTN*-KO rabbits using the CRISPR/Cas9 system

In the present study, a total of 48 injected zygotes were transferred into the oviducts of three surrogate rabbits. After full-term gestation, the three surrogate mothers successfully gave birth to 12 live pups (Table 1).

**Table 1:** Summary of the production of *MSTN*-KO rabbits using CRISPR/Cas9

Targeting vector	gRNA/Cas9 mRNA (ng/µl)	Embryos transferred	Pups obtained (% transferred)	Mutation efficiency (% pubs)	Biallelic-KO efficiency (% pubs)
sgRNA-1	10/40	20	7/20 (35.00%)	7/7 (100%)	7/7 (100%)
sgRNA-2	10/40	28	5/28 (17.85%)	4/5 (80.00%)	4/4 (100%)

**Figure 2:**
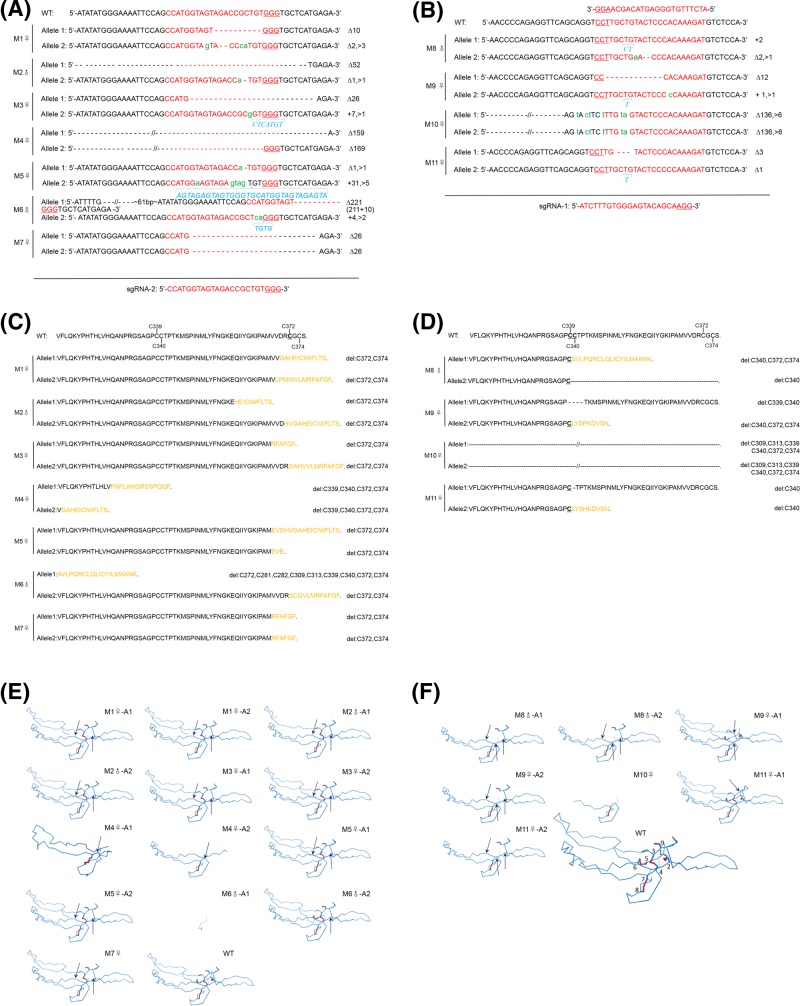
Mutations of *MSTN* induced by CRISPR/Cas9 (**A**) Sequences obtained from mutant rabbits following microinjection of Cas9 mRNA and sgRNA-2. (**B**) Sequences obtained from mutant rabbits generated following the microinjection of Cas9 mRNA and sgRNA-1. The DNA sequences for which the sgRNAs were designed are highlighted in red, and PAM sequences are highlighted with an underline. The WT sequence is shown at the top of the mutant sequence. Insertions (+) and deletions (Δ) are shown to the right of each allele. Substitutions (>) are labeled in green lowercase, and insertions are labeled in blue italicized letters. (**C**) The predicted partial amino acid sequences of the founder rabbits mediated by sgRNA2. C372 is highlighted with an underline, and cysteine deletions (Del) are shown to the right of each amino acid sequence. (**D**) The predicted partial amino acid sequences of the founder rabbits mediated by sgRNA1. C339 and C340 are highlighted with an underline, and cysteine deletions (Del) are shown to the right of each amino acid sequence. (**E**) The tertiary structures of the modified alleles targeted by sgRNA2. (**F**) The tertiary structures of the modified alleles targeted by sgRNA1. The tertiary structures are partly shown in the pictures above (from amino acid 263 to 375). The disulfide bond is highlighted in red, and disulfide deletions are marked by black arrows. WT: 1: C374, 2: C313, 3: C340, 4: C287, 5: C372, 6: C309, 7: C282, 8: C272, and 9: C339. Abbreviation: PAM, protospacer adjacent motif.

**Figure 3:**
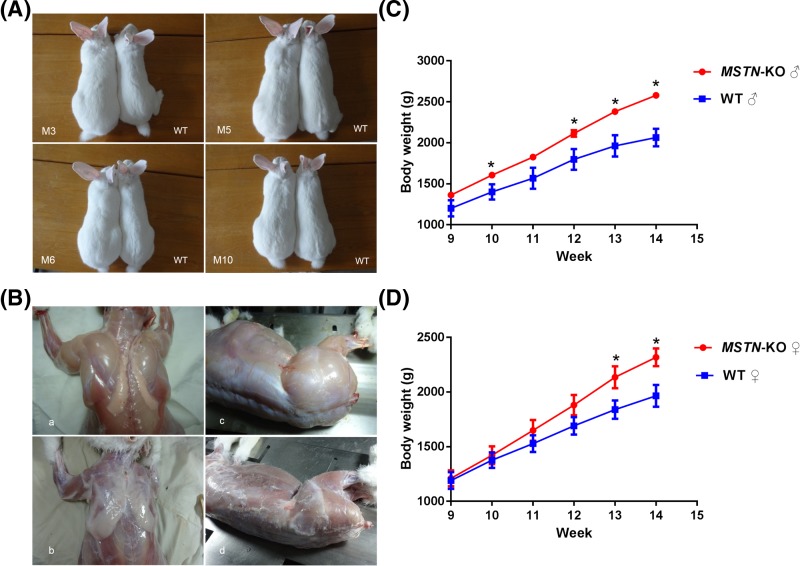
Photos of the *MSTN*-KO and WT rabbits (**A**) A comparison of the *MSTN*-KO and WT rabbits. WT: wild-type rabbit; M3, M5, M6, and M10: *MSTN*-KO rabbits. (**B**) Muscle maps of the *MSTN*-KO rabbit. (a) Arm and rear muscle of the *MSTN*-KO rabbit. (b) Arm and rear muscle of the WT rabbit. (c) Hip muscle of the *MSTN*-KO rabbit. (d) Hip muscle of the WT rabbit. (**C**) Changes in the average body weight of the *MSTN*-KO male rabbits (*n*=3) and the WT male rabbits (*n*=5) from 9 to 14 weeks of age. (**D**) Changes in the average body weight of the *MSTN*-KO female rabbits (*n*=5) and the WT female rabbits (*n*=5) from 9 to 14 weeks of age. Mean ± S.E.M., *P<0.05.

**Figure 4:**
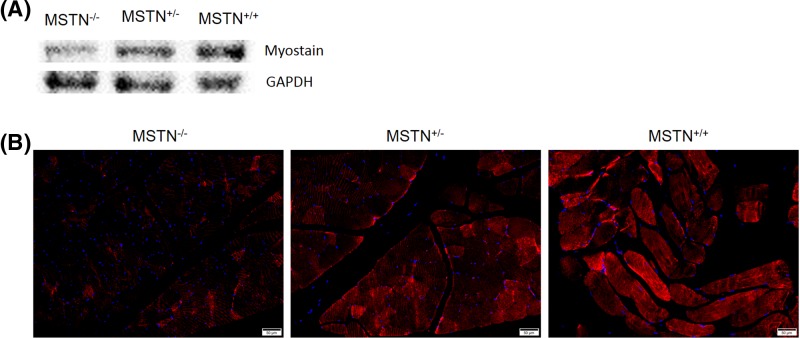
Expression of MSTN protein in skeletal muscle (**A**) Detection of MSTN protein in gluteus maximus tissue using Western blot analysis. GAPDH was used as a reference control. (**B**) Detection of MSTN protein in gluteus maximus tissue using immunofluorescence staining. Scale bar = 50 μm.

Genomic DNA was extracted from the ear tissue of the newborn pups. The 12 individuals used for genotyping were numbered M1−M12. The T-cloning and PCR-sequence results showed that the *MSTN* mutation was detected in 11 pups, and the indels ranged from 1 to 221 bp (Figures 1C and 2A,B). The theoretical amino acid sequences of the *MSTN* knockout alleles are shown in Figure 2C,D. In addition, fragment deletions between the two sgRNAs targeting sites were frequently observed in the present study. Here, only one rabbit (M10♀) harbored mutations in exon 3 of the *MSTN* gene that did not cause a frameshift mutation. Eleven rabbits (M1−M11) deleted C372 and C374, and four rabbits (M8−M11) deleted C339 and C340 in two alleles, C281–C340. M7♀ and M10♀ were homozygous, and both of them lost C372 and C374 in the cystine-knot motif. In addition, M10♀ also deleted C339 and C340. As shown in Figure 2E,F, the structure of each protein differs from the WT for the cysteine delete (like C339, C340, C372, and C374). Five rabbits (M1♀, M2♂, M3♀, M5♀, and M7♀) deleted C272, C281, C282, C339, and C340. In the cysteines mentioned above, four cysteines formed two intramolecular disulfide bonds (C272−C282, C281−C340), and C339 was involved in intermolecular disulfide bond formation between two *MSTN* monomers. The typical phenotype of double-muscling was observed in F0 *MSTN*-KO rabbits at four months of age, and they were compared with their WT counterparts, as shown in Figure 3A,B. To investigate whether gene mutations abolished the MSTN protein translation, Western blotting and immunofluorescence staining were performed. As shown in Figure 4, the MSTN protein was significantly decreased in *MSTN^−/−^* and *MSTN^+/−^* rabbits compared with *MSTN^+/+^* counterparts.

### Increased body weights in the *MSTN*-KO rabbits

Rabbits experience an exuberant growth period from 2 to 3 months, so the body weights of the *MSTN*-KO rabbits and their WT counterparts were recorded from 9 to 14 weeks. It was found that the average body weights of male *MSTN*-KO rabbits were significantly higher than the male WT rabbits from 9 to 14 weeks of age (Figure 3C). The average body weights of female *MSTN*-KO rabbits were significantly higher than the female WT rabbits from 11 to 14 weeks of age (Figure 3D). Significant differences (*P<0.05) from 10 to 14 weeks after birth were found between the *MSTN*-KO male rabbits and the WT male rabbits. Significant differences (*P<0.05) were found between the *MSTN*-KO female rabbits and the WT female rabbits from 13 to 14 weeks after birth. At 14 weeks, the average body weights of the female (2318.40 ± 183.15 g) and male (2579.33 ± 67.99 g) *MSTN*-KO rabbits increased by 17.91 and 26.74%, respectively, compared with the female (female: 1966.20 ± 225.56 g) and male (2063.33 ± 186.47 g) WT rabbits, respectively.

### Heritability and breeding of the *MSTN*-KO rabbits

The male founders (M2, M6, and M8) were mated with the female WT rabbits, and the female founders (M1, M3, M5, and M10) were mated with the male WT rabbits. Genomic DNA was extracted from the ear tissues of the F1 rabbits. PCR amplification and TA-cloning sequencing analysis showed that these F1 rabbits were monoallelic *MSTN*-KO rabbits. Then the same genotype of the F1 male and female rabbits was mated for homozygous mutants. The breeding results are shown in Supplementary Table S1.

### Off-target assay

A total of ten potential off-target (OT) sites (OTs, five for sgRNA1 and five for sgRNA2) were successfully amplified using PCR and subjected to Sanger sequencing. No overlapping peaks were detected near the OTs.

### Histological analysis

**Figure 5:**
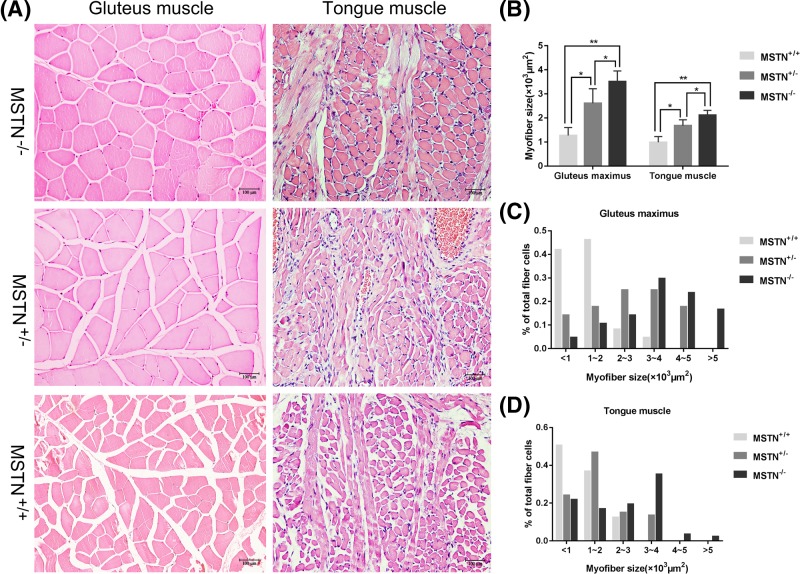
Histological analysis (**A**) Hematoxylin and Eosin-stained cross-sections of the gluteus and tongue muscles from *MSTN*-KO rabbits and WT rabbits. Scale bar = 100 μm. (**B**) Average size and density of myofibers in the gluteus and tongue muscles. The relative size of myofibers in the gluteus from WT rabbits (*n*=5) and *MSTN*-KO rabbits (*n*=5) and the tongue from WT rabbits (*n*=5) and *MSTN*-KO rabbits (*n*=5). Mean ± S.E.M.; *P<0.05 and **P<0.01. (**C**) Distribution of different sizes of myofibers in gluteus muscles from WT rabbits and *MSTN*-KO rabbits. Samples were collected from 6-month-old rabbits. (**D**) Distribution of different sizes of myofibers in the tongue muscles from WT rabbits and *MSTN*-KO rabbits. Samples were collected from 6-month-old rabbits.

The *MSTN*-KO rabbits exhibited the double-muscled phenotype (Figure 3A), and a histological examination of the gluteus maximus and tongue showed muscle fiber hypertrophy relative to the fibers of the WT rabbit (Figure 5A). In Figure 5B, the average size of the gluteus maximus myofibers in the *MSTN^−/−^* rabbits (3512.20 ± 439.17 μm^2^) was substantially larger than that of the *MSTN^+/+^* rabbits (1274.76 ± 327.30 μm^2^, *P*<0.01). The average size of the gluteus maximus myofibers in the *MSTN^+/−^* rabbits (2610.37 ± 604.44 μm^2^) increased in comparison with the *MSTN^+/+^* rabbits (1274.76 ± 327.30 μm^2^, *P*<0.05). Similarly, the average size of tongue myofibers in the *MSTN^−/−^* rabbits (2109.22 ± 206.75 μm^2^) was substantially larger than that of the *MSTN^+/+^*rabbits (989.39 ± 239.27 μm^2^, *P*<0.01). The average size of tongue myofibers in the *MSTN^+/−^* rabbits (1677.24 ± 247.50 μm^2^) increased in comparison with the *MSTN^+/+^* rabbits (989.39 ± 239.27 μm^2^, *P*<0.05). The distribution of different sizes of gluteus maximus and tongue myofibers indicates that the percentage of smaller fiber cells in the *MSTN*-KO rabbits was lower than the percentage in the WT rabbits (Figure 5C,D).

### Analysis of hematological and biochemical parameters

**Figure 6:**
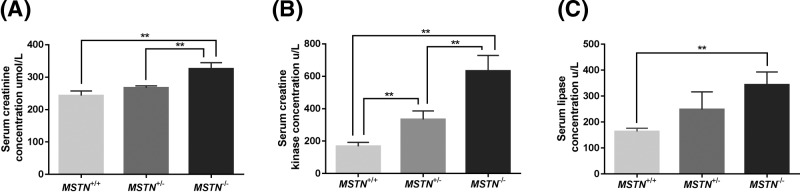
Plasma levels of the *MSTN*-KO and the WT rabbits at 6 months of age (**A**) Plasma levels of CREA of the *MSTN*-KO rabbits and the WT rabbits. (**B**) Plasma levels of CK of the *MSTN*-KO rabbits and the WT rabbits. (**C**) LIP plasma levels of the *MSTN*-KO and WT rabbits. Mean ± S.E.M.; **P<0.01.

The hematological and biochemical parameters from 6-month-old *MSTN^+/+^, MSTN*^+/−^, and *MSTN*^−/−^ rabbits were analyzed. In Figure 6, the levels of serum creatinine (CREA, 325.97 ± 18.92 μmol/l), serum creatine kinase (CK, 599.67 ± 13.79 U/l), and serum lipase (LIP, 343.17 ± 49.99 U/l) were increased in the *MSTN*^−/−^ rabbits compared with the *MSTN*^+/+^ rabbits (CREA, 243.17 ± 14.35 μmol/l; CK, 167.67 ± 24.83 U/l; LIP, 163.70 ± 12.24 U/l, *P*<0.01). The levels of CREA (325.97 ± 18.92 μmol/l) and CK (599.67 ± 13.79 U/l) were increased in the *MSTN*^−/−^ rabbits compared with the *MSTN*^+/−^ rabbits (CREA, 267.10 ± 6.06 μmol/l; CK, 333.67 ± 52.54 U/l, *P*<0.01). The levels of CK (CK, 333.67 ± 52.54 U/l) were increased in the *MSTN*^+/−^ rabbits compared with the *MSTN*^+/+^ rabbits (CK, 167.67 ± 24.83 U/l, *P*<0.01). CREA is a product of muscle tissue metabolism in the body. Serum CK is related to the total amount of muscle. This means that the greater the muscle capacity, the higher the serum CK activity. Increased serum CREA and CK levels in the MSTN-KO rabbits should correlate with increased muscle mass. The serum CREA content reported for *MSTN*-KO Meishan pigs was also higher than that in WT pigs [25]. Interestingly, serum LIP was greater in the *MSTN*^−/−^ rabbits than that in the *MSTN*^+/+^ rabbits at 6 months of age. Further experiments are required to investigate this phenomenon in *MSTN*^−/−^ rabbits. There were no differences in the hematology characteristics and other biochemical parameters. The results are listed in Supplementary Tables S2 and S3.

### Effect of *MSTN* mutation on the teeth and pelvis in *MSTN*-KO rabbits

**Figure 7:**
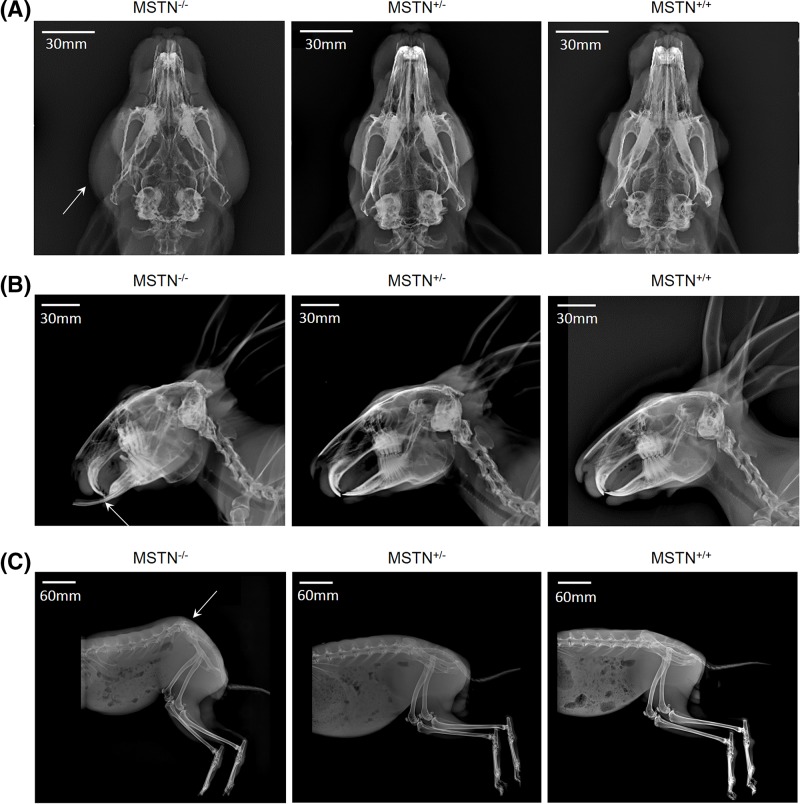
Radiographs of *MSTN*-KO and WT rabbits (**A**) Radiographs of the skull from the basilar aspect in the *MSTN*-KO and WT rabbits showing the masseter muscles (white arrow) in the *MSTN*^−/−^ rabbit. (**B**) Radiographs of the teeth from the lateral aspect in the *MSTN*-KO and WT rabbits showing the teeth dislocation (white arrow) in the *MSTN*^−/−^ rabbit. (**C**) Radiographs of the rear quarters from the lateral aspect in the *MSTN*-KO and WT rabbits in a state of natural relaxation showing the pelvic tilt in the rabbits.

To study if there were any effects of the targeted *MSTN* mutation on the rabbits, the body structure and physiological activity of the *MSTN*-KO rabbits were closely observed. It was found that the *MSTN*^−/−^ rabbits showed a greater mass of the masseter muscles compared with the WT and *MSTN*^+/−^ rabbits (Figure 7A). In addition, teeth dislocation and pelvic tilt were present in the *MSTN*^−/−^ rabbits at 3−6 months of age. All of the biallelic *MSTN*-KO rabbits showed tongue enlargement at birth. A statistical analysis of the teeth dislocation and pelvic tilt in all three genotypes of the *MSTN*-KO rabbits, including 88 *MSTN*^+/+^, 77 *MSTN*^+/−^, and 17 *MSTN*^−/−^, showed that the percentage of rabbits having teeth dislocation was 0% in *MSTN*^+/+^, 0% in *MSTN*^+/−^, and 100% in *MSTN*^−/−^ rabbits. The percentage of rabbits having pelvic tilt was 0% in *MSTN*^+/+^, 0% in *MSTN*^+/−^, 77.78% in female *MSTN*^−/−^ rabbits, and 37.50% in male *MSTN*^−/−^ rabbits (Figure 7B,C). Teeth dislocation and pelvic tilt were not present among the WT and *MSTN*^+/−^ rabbit population in the laboratory. Teeth dislocation and pelvic tilt are likely the effects of the targeted *myostatin* allele mutation in rabbits. These results imply that over-developed muscles have an effect on bone morphology.

## Discussion

In the present study, the *MSTN*-KO rabbits showed a ‘double-muscling’ phenomenon and a dramatic increase in body weight. At the end of 14 weeks, the *MSTN*-KO rabbits exhibited an approximately 30% increase in body weight compared with WT rabbits that might be attributed to the *MSTN* gene mutation factor, irrelevant to age and sex. These results are consistent with the *MSTN*-KO mice produced by the homozygous deletion of the C-terminal region of the *myostatin* gene [2,8,26], indicating that disruption of cystine-knot motif can increase muscle mass. This also shows that the structure of the cystine-knot motif plays an important role in myostatin activity. An intricate cysteine-knot motif contains nine cysteine residues (C272, C281, C282, C309, C313, C339, C340, C372, and C374) in the C-terminal region of mammalian myostatin protein [27]. The nine cysteine residues form disulfide bonds that are crucial for the formation of three-dimensional protein structures and could influence the function of other motifs (such as ligand binding or signaling) in the same protein [28,29]. Disruption of the cystine-knot and decreased covalent dimerization likely results in a less compact structure that is then unsuitable for type I and type II Ser/Thr kinase transmembrane receptors activation [14,30]. An analysis of the C313A and C374A mutations confirmed that it is the disruption of the cystine-knot by removal of the C313–C374 disulfide bond, and not the introduction of the tyrosine, that is the dominant factor in the decreased stability and reduced disulfide-linked dimerization of C313Y myostatin [14]. This means that removing the disulfide bonds destroys the cystine-knot motifs. Various mutational *MSTN* genes of the 11 founder rabbits were modeled using the SWISS-MODEL, and the cystine-knot motifs were disrupted, leading to disulfide bond loss and great changes in the tertiary structures. Furthermore, as this research progresses and more offspring are produced, this *MSTN*-KO model can be used to incorporate more reliable parameters and may offer insights into the mechanisms that control signaling in skeletal muscle.

In the present study, all of the homozygous mutant rabbits showed pelvic tilt, teeth dislocation, and tongue enlargement. This OT phenomenon was not found in the *MSTN*^−^*MSTN*-KO rabbits [24,31] and *MSTN*-KO pigs [21]. Previous studies have demonstrated that an enlarged tongue is primarily attributed to muscle fiber hypertrophy [24]. However, the teeth dislocation and pelvic tilt phenomena have rarely been reported in other *MSTN*^−/−^ animals. Myostatin is expressed only in muscle and not in normal bone or other connective tissue [8]. It has long been recognized that increased muscle mass and strength are associated with a resultant increase in bone mass [32–34]. Studies have shown that MSTN has an effect on the bone mass and bone volume fraction in mice [35]. Byron et al. [36] and Vecchione et al. [37] employed a hypermuscular mouse model (mice lacking myostatin) to explore the effects of increased muscle mass. They reported that adult *MSTN*-KO mice were generally more brachycephalic, had a smaller cranial vault and maxillary lengths, and exhibited significantly different mandibular shapes, with the mandible longer and shortened in the vertical dimension compared with WT controls. Byron et al. [16] further revealed that increasing masticatory muscle mass and bite force would increase sagittal suture waveform complexity based on research of *MSTN*-KO mice. Recently, a study on *MSTN*-KO Meishan pigs found that 20% *MSTN*-KO Meishan pigs had one extra thoracic vertebra, and the researchers demonstrated that this phenomenon was caused by the *MSTN* mutation [25]. To our knowledge, the effect of *MSTN* deficiency on rabbit teeth and the pelvis has rarely been reported in other *MSTN*-KO animals. Statistically significant differences have been found in joint proportions and bone mineral densities between normal and *MSTN*-KO mice [20]. Moreover, a recent study has shown that mice homozygous for the myostatin mutation had increased muscle mass and craniofacial dysmorphology in adulthood [3]. Significant correlations have also been noted between masseter muscle weight and mandibular body length [37]. In this study, the teeth dislocation phenomenon may have been due to masticatory hypermuscularity that resulted in significantly altered craniofacial morphology, likely due to altered biomechanical stress. Rabbits excel at sprinting and jumping due to short forelimbs and long hind legs. In *MSTN*^−/−^ rabbits, the elevated hindquarter muscle mass and physiological cross-section enhanced jumping forces compared with similar-sized *MSTN^+/^*^−^ rabbits. Following alterations in the local biomechanical conditions, the elevated hindquarter muscle loads led to an alteration in the morphology of the pelvis in *MSTN*^−/−^ rabbits. These findings demonstrate that muscle function plays an important role in bone morphology. It was found in this study that *MSTN^+/−^* rabbits displayed mild phenotypes and mild abnormalities. This result is consistent with studies of both *MSTN^+/^*^−^ mice and *MSTN^+/^*^−^ cattle, and the abnormalities were likely caused by haploinsufficiency [4]. In these allelic mutation rabbits, it was found that the incidence of pelvic tilt in female *MSTN^−/−^* rabbits (77.78%) was significantly higher than that in male *MSTN^−/−^* rabbits (37.50%). The conclusion drawn was that this may be due to the difference in the pelvic structures of males and females. In mammals, the pelvis is significantly larger in females than in males to allow babies to pass through pelvis when they are born. However, it was still unclear if these craniofacial and pelvic tilt abnormalities were related to the *MSTN* knockout condition during the pre- or neonatal period or were simply a result of postnatal compensatory changes. The effect of the molecular mechanism of the loss of myostatin function on the craniofacial and pelvic tilt abnormalities in rabbits needs further investigation.

The *MSTN*-KO mouse has typically been used as a new animal model for the study of the relationship among craniofacial morphology, hypermuscularity, and increased bite force since its muscles have been demonstrated to be significantly larger [16,38]. In a clinical setting, non-specific chronic low back pain (cLBP) is associated with pelvic tilt [39]. It can be concluded that the *MSTN*-KO rabbits will be a better model for pelvic tilt correction and craniofacial research due to their observable pelvis and skulls, appropriate body, longer life cycles, and easy management due to low feeding costs.

## Conclusion

In conclusion, *MSTN*-KO rabbits were generated with remarkably high efficiency by microinjecting Cas9 mRNA and sgRNA into pronuclear-stage embryos. The results of the present study suggest that Cas9 mRNA microinjection may provide a fast and efficient approach for gene knockout rabbit models. Although muscle overgrowth caused by homozygous mutations in *myostatin* was impressive, bone abnormalities caused by excessively growing muscles may be a health hazard for livestock. In addition, based on the abnormality results found in *MSTN*-KO rabbits, it is suggested that these rabbits would make an important animal model for craniofacial and pelvic research.

## Materials and methods

### Animals and ethics statement

The New Zealand rabbits used in the present study were kept at the Animal Genetic Engineering Laboratory of Yangzhou University. They were fed twice a day and offered water limitlessly. Animal experiments and procedures were performed in accordance with the Guide for the Care and Use of Laboratory Animals (Ministry of Science and Technology of the People’s Republic of China) and approved by the Animal Care and Use Committee of Yangzhou University, Yangzhou, China (license number: SYXK(Su)2017-0044).

### CRISPR/Cas9 expression plasmids construction and *in vitro* transcription

We constructed two CRISPR/Cas9 expression plasmids that were targeted to the cystine-knot motif. To ensure successful targeting, two sgRNAs independently targeting exon 3 of *MSTN* (sgRNA1 sited E3: g.4881-4903 and sgRNA2 sited E3: g.4796-4818) were performed, as shown in Figure 1B.

To construct the recombinant vector for the preparation of sgRNA by *in vitro* transcription, a pair of complementary DNA oligos, shown in Supplementary Table S4, were annealed to be double-stranded. They were then subcloned into the linearized PYSY-T7-Cas9-T7-MSTN-gRNA cloning vector, which was provided by the Nanjing YSY Biotech Com., Ltd., Nanjing, China. The PCR product for the *in vitro* transcription of Cas9 mRNA was amplified using T7 primers (T7-1:5′-TGTAAAACGACGGCCAGT-3′ and T7-2: 5′-TGGCACCGAGTCGGTGCTTT -3′). The PCR products for the *in vitro* transcription of sgRNA were amplified using T7 primers (T7-3: 5′-ATTAACCCTCACTAAAGGGA-3′ and T7-4: 5′-AAAAAAAGCACCGACTCGGTGCCAC-3′). Cas9 mRNA and sgRNAs were transcribed *in vitro* using the ScriptMAX Thermo T7 Transcription Kit (TSK-101, Toyobo, Japan) and purified using a lithium chloride precipitation solution (AM9480, Ambion, U.S.A.) according to the manufacturer’s instructions. The concentration and quality of the synthesized mRNA were determined using the spectrophotometer (OD-1000+, Nanjing Wuyi technology Com., Ltd., Nanjing, China) and agarose gel electrophoresis, respectively. Cas9 mRNA and sgRNAs were diluted into RNase-free water and stored at −80°C for future use.

### Zygote injection with Cas9/sgRNA

Healthy donors with regular estrus cycles were selected for zygote collection. Zygotes were collected using surgical oviduct flushing from donors after superovulation treatment and natural mating. In brief, donors were injected with follicle stimulating hormone (FSH) for estrus synchronization. A total of 60 IU FSH were administered by intramuscular injection in six dosages at 12-h intervals (the first dose was 15 IU and other doses decreased progressively to 5 IU). The animals were then injected with 100 IU of HCG 12 h after the FSH was administered. The females were then subsequently mated with a male rabbit.

Rabbit embryos at the pronuclear stage (approximately 18–20 h post-mating) were transferred into M2 medium (M7167, Sigma, U.S.A.) with a 1% penicillin–streptomycin solution (SV30010, HyClone, U.S.A.) and 10% fetal bovine serum (FBS, SH30084.03, HyClone, U.S.A.). A mixture of *in vitro* transcribed sgRNA (10 ng/μl) and Cas9 mRNA (40 ng/μl) was injected into the cytoplasm of pronuclear stage embryos. The injected embryos were transferred to M16 medium (M7292, Sigma, U.S.A.) with a 1% penicillin–streptomycin solution and 10% FBS for a 30–60 min culture at 38.5°C, 5% carbon dioxide, and humidity conditions. Then approximately 15−20 injected embryos were transferred into the oviducts of the recipient mother.

### DNA extraction, PCR, and sequencing

The genomic DNA of the sample was extracted from a small piece of ear biopsy using the phenol–chloroform extraction method. The target sites were PCR amplified using the primers (CAS9SG12-1 and CAS9SG12-2). The sequences of primers are shown in Supplementary Table S5. The PCR products were subjected to 1% agarose gel electrophoresis, and the map is shown in Figure 1C. To test the mutation patterns, the PCR products were purified using a PCR Purification kit (EP101-01, TransGen, China) and cloned into the pGEM-T vector (A1360, Promega, U.S.A.). A total of 50 positive T-clones were sequenced by the Beijing Genomics Institute. Lasergene (DNASTAR, Inc., U.S.A.) was used for the sequence analysis. The tertiary structure of the theoretical amino acid sequences was modeled using SWISS-MODEL (https://www.swissmodel.expasy.org/).

### Western blotting and immunofluorescence analysis

Total protein was extracted from gluteus maximus tissue in *MSTN*^+/+^, *MSTN*^+/−^, and *MSTN*^−/−^ rabbits using radio-immunoprecipitation assay (RIPA) lysis buffer (P0013B, Beyotime, China) with protease inhibitors according to the manufacturer’s instructions. The protein concentrations were determined using the bicinchoninic assay (BCA) method (CW0014S, CWBIO, China). A 40-μg protein sample was subjected to a 12% polyacrylamide Tris-glycine gel for 1 h. Separated proteins were then transferred to polyvinylidene difluoride (PVDF) membranes (F019531, Sangon, China). The membranes were then blocked for 2 h in a Tris buffered saline with Tween 20 (TBST) buffer containing 5% milk at room temperature. The membranes were subsequently incubated for 1.5 h at 37°C with primary monoclonal antibodies against MSTN (sc-393335, Santa Cruz Biotechnology, U.S.A.) diluted to 1:100 with TBST buffer. The membranes were subsequently washed three times for five minutes with the TBST buffer and incubated with anti-mouse secondary antibodies (D110096, Sangon, China) for 1.5 h at 37°C. The GAPDH antibody (CW0100M, CWBIO, China) was used as a reference control. The proteins were visualized with the ECL substrate solution (#170-5060, Bio-Rad, U.S.A.) using an imaging system (5200, Tanon, China).

Gluteus maximus tissue from *MSTN*^+/+^, *MSTN*^+/−^, and *MSTN*^−/−^ rabbits were collected and fixed with 4% paraformaldehyde, followed by embedding in paraffin according to routine protocol. Deparaffinized tissues were sectioned at 4-μm thicknesses and blocked in 5% bovine serum albumin (BSA). Sections were stained with a primary monoclonal antibody against MSTN (sc-393335, Santa Cruz Biotechnology, U.S.A.), and diluted to 1:50 with a blocking buffer by incubating at 4°C overnight. After washing, sections were incubated with secondary antibodies (BA1105, Boster, China) for 1 h at 37°C and then for an additional 5 min with 4,6-diamidino-2-phenylindole (C1002, Beyotime, China). Images were taken using a microscope (BX53, Olympus, Japan).

### Increased body weights in *MSTN*-KO rabbits

In the preent study, the *MSTN*-KO founder rabbits and WT counterpart controls were bred and housed under the same conditions. Their feed was adjusted with respect to growth stages after weaning at 30 days of age. Their body weights were recorded daily from 9 to 14 weeks of age.

### OT assay

To determine the site-specific cleavage of the CRISPR-Cas9 system *in vivo*, all possible sites of the entire rabbit genome with homology to the 23-bp sequence (sgRNA + PAM) were predicted using the CRISPR design tool (http://tools.genomeengineering.org). The mismatch parameter for the target sequence was set as 5′–NGG and was chosen as the protospacer adjacent motifs (PAM). The sites with 12-bp conserved proximal to PAM and total mismatches <4 and sites with total mismatches <5 were chosen as potential OT sites for subsequent testing. The selected potential OT sites were initially PCR-amplified using genomic DNA from *MSTN*-KO rabbits. The PCR products were then used for sequencing. The information of the OT sites and primer pairs used is listed in Supplementary Tables S6 and S7.

### Histology analysis

Tissues from the gluteus maximus muscles and tongues of an *MSTN*^−/−^ and a WT rabbit of the same age and anatomical position were collected and fixed in 4% paraformaldehyde. These tissues were dehydrated in a graded alcohol series and then embedded in paraffin wax and slide sectioned at 3–5 μm. The slide sections were then stained with Hematoxylin and Eosin (HE) and viewed using a microscope (Leica, DM2000, Germany). For each sample, five fields of view (areas) were randomly selected in the HE-stained sections using a 200× objective and then analyzed using Image-Pro Plus 6.0 software (Media Cybernetics, Inc., U.S.A.). For each sample area, 100–150 myofibers were measured, and the relative size of the myofibers and the distribution of different sizes of the myofibers were determined.

### Analysis of hematological and biochemical parameters

Blood samples were obtained from rabbits that had been fasted for 12 h, and ethylenediaminetetraacetic acid (EDTA)-plasma and plasma samples were collected after centrifugation at 4°C for 20 min.

An automatic biochemical analyzer (AU480, Beckman Coulter, Inc., U.S.A.) was used to test the concentration of alanine aminotransferase (ALT), aspartate aminotransferase (AST), alkaline phosphatase (ALP), lactate dehydrogenase (LDH), creatine kinase (CK), creatinine (CREA), glucose (GLU), total cholesterol (TC), triglycerides (TG), calcium (CA), phosphorus (P), and LIP with commercial kits (PT9175, OT9176, AP9209, LD9178, CK9038, CR9169, GL9158, CH9192, TG9179, CA8173, PH9136, and LI9068. Ningbo Purebio Biotech Com., Ltd., Ningbo, China). SPSS13.0 software was used to analyze the measured results. Hematology parameters, like RBC, HGB, HCT, MCV, MCH, MCHC, WBC, RDW-CV, PLT, and MPV, were tested using an auto-hematology analyzer (BC-2800, Mindary Biotech Com., Ltd., Shenzhen, China).

### Statistical analysis

Statistical comparisons of body weight, myofiber size, and blood parameters among the *MSTN*-KO and WT rabbits were performed using a Student’s *t* test, and all data were expressed as a mean ± S.E.M. Statistical analyses were conducted using GraphPad Prism software, and *P*<0.05 was considered as statistically significant (*P<0.05, **P<0.01, ***P<0.001).

## Availability of data and materials

The data supporting the results of this manuscript are included in the body of the manuscript and as Supplementary data.

## Supporting information

**Supplemental Table S1:** Summary of heritability and breed of the MSTN-KO rabbits

**Supplemental Table S2:** Hematology parameters of the *MSTN*^+/+^, *MSTN*^+/-^ and *MSTN*^-/-^ rabbits.

**Supplemental Table S3:** Biochemical parameters of the *MSTN*^+/+^, *MSTN*^+/-^ and *MSTN*^-/-^ rabbits.

**Supplemental Table S4:** The sequences of complementary DNA oligos.

**Supplemental Table S5:** Primers for detection of *MSTN*-KO rabbits.

**Supplemental Table S6:** The sequences of potential off-target sites.

**Supplemental Table S7:** The sequences of potential off-target loci PCR primers.

## Abbreviations

CK: creatine kinase
CREA: creatinine
FBS: fetal bovine serum
FSH: follicle stimulating hormone
GAPDH: Glyceraldehyde-phosphate dehydrogenase
HCG: Human Chorionic Gonadotropin
HE: Hematoxylin and Eosin
KO: Knockout
LIP: lipase
MSTN: myostatin
nt821(del11): 11-bp deletion at the bovine *myostatin* nucleotide 821
OT: off-target
PAM: protospacer adjacent motif
sgRNA: small guide RNA
TBST: Tris buffered saline with Tween 20
TGF-β: transforming growth factor β
WT: wild-type

## References

[1] Lee S.J. (2004) Regulation of muscle mass by myostatin. Annu. Rev. Cell Dev. Biol. 20, 61–86 10.1146/annurev.cellbio.20.012103.135836 15473835

[2] McPherron A.C., Lawler A.M. and Lee S.J. (1997) Regulation of skeletal muscle mass in mice by a new TGF-beta superfamily member. Nature 387, 83–90 10.1038/387083a0 9139826

[3] Vecchione L. (2010) Age-related changes in craniofacial morphology in GDF-8 (myostatin)-deficient mice. Anat. Rec. (Hoboken) 293, 32–41 10.1002/ar.21024 19899116 PMC3113544

[4] Kambadur R. (1997) Mutations in myostatin (GDF8) in double-muscled Belgian Blue and Piedmontese cattle. Genome Res. 7, 910–916 10.1101/gr.7.9.910 9314496

[5] Mosher D.S. (2007) A mutation in the myostatin gene increases muscle mass and enhances racing performance in heterozygote dogs. PLoS Genet. 3, e79 10.1371/journal.pgen.0030079 17530926 PMC1877876

[6] Schuelke M. (2004) Brief report - Myostatin mutation associated with gross muscle hypertrophy in a child. N. Engl. J. Med. 350, 2682–2688 10.1056/NEJMoa040933 15215484

[7] Boman I.A. (2009) A frameshift mutation in the coding region of the myostatin gene (MSTN) affects carcass conformation and fatness in Norwegian White Sheep (*Ovis aries*). Anim. Genet. 40, 418–422 10.1111/j.1365-2052.2009.01855.x 19392824

[8] McPherron A.C. and Lee S.-J. (1997) Double muscling in cattle due to mutations in the myostatin gene. Proc. Natl. Acad. Sci. U.S.A. 94, 12457–12461 10.1073/pnas.94.23.12457 9356471 PMC24998

[9] Grobet L. (1997) A deletion in the bovine myostatin gene causes the double-muscled phenotype in cattle. Nat. Genet. 17, 71–74 10.1038/ng0997-71 9288100

[10] Grobet L. (1998) Molecular definition of an allelic series of mutations disrupting the myostatin function and causing double-muscling in cattle. Mamm. Genome 9, 210–213 10.1007/s003359900727 9501304

[11] Jeanplong F. (2001) Genomic organization and neonatal expression of the bovine myostatin gene. Mol. Cell. Biochem. 220, 31–37 10.1023/A:1010801511963 11451380

[12] Berry C. (2002) Single cysteine to tyrosine transition inactivates the growth inhibitory function of Piedmontese myostatin. Am. J. Physiol. Cell Physiol. 283, C135–C141 10.1152/ajpcell.00458.2001 12055081

[13] Cash J.N. (2009) The structure of myostatin: follistatin 288: insights into receptor utilization and heparin binding. EMBO J. 28, 2662–2676 10.1038/emboj.2009.205 19644449 PMC2738701

[14] Starck C.S. and Sutherland-Smith A.J. (2011) The C313Y Piedmontese mutation decreases myostatin covalent dimerisation and stability. BMC Res. Notes 4, 442 10.1186/1756-0500-4-442 22023879 PMC3213697

[15] Grobet L. (1998) Molecular definition of an allelic series of mutations disrupting the myostatin function and causing double-muscling in cattle. Mamm. Genome 9, 210–213 10.1007/s003359900727 9501304

[16] Byron C.D. (2004) Effects of increased muscle mass on mouse sagittal suture morphology and mechanics. Anat. Rec. A Discov. Mol. Cell Evol. Biol. 279, 676–684 10.1002/ar.a.20055 15224409

[17] Williams S.H. (2015) Effect of postnatal myostatin inhibition on bite mechanics in mice. PLoS ONE 10, e0134854 10.1371/journal.pone.0134854 26252892 PMC4529299

[18] Jeffery N. and Mendias C. (2014) Endocranial and masticatory muscle volumes in myostatin-deficient mice. R. Soc. Open Sci. 1, 140187 10.1098/rsos.140187 26064569 PMC4448778

[19] Byron C.D. (2008) Enlargement of the temporalis muscle and alterations in the lateral cranial vault. Integr. Comp. Biol. 48, 338–344 10.1093/icb/icn020 21669796

[20] Ravosa M.J. (2008) Using “Mighty Mouse” to understand masticatory plasticity: myostatin-deficient mice and musculoskeletal function. Integr. Comp. Biol. 48, 345–359 10.1093/icb/icn050 21669797

[21] Wang K. (2015) Efficient generation of myostatin mutations in pigs using the CRISPR/Cas9 system. Sci. Rep. 5, 16623 10.1038/srep16623 26564781 PMC4643223

[22] He Z. (2018) Use of CRISPR/Cas9 technology efficiently targetted goat myostatin through zygotes microinjection resulting in double-muscled phenotype in goats. Biosci. Rep. 38, BSR20180742 10.1042/BSR20180742 30201688 PMC6239268

[23] Crispo M. (2015) Efficient generation of myostatin knock-out sheep using CRISPR/Cas9 technology and microinjection into zygotes. PLoS ONE 10, e0136690 10.1371/journal.pone.0136690 26305800 PMC4549068

[24] Lv Q. (2016) Efficient generation of myostatin gene mutated rabbit by CRISPR/Cas9. Sci. Rep. 6, 25029 10.1038/srep25029 27113799 PMC4844959

[25] Qian L. (2015) Targeted mutations in myostatin by zinc-finger nucleases result in double-muscled phenotype in Meishan pigs. Sci. Rep. 5, 14435 10.1038/srep14435 26400270 PMC4585837

[26] McPherron A.C., Huynh T.V. and Lee S.J. (2009) Redundancy of myostatin and growth/differentiation factor 11 function. BMC Dev. Biol. 9, 24 10.1186/1471-213X-9-24 19298661 PMC2666675

[27] Carnac G. (2006) Myostatin: biology and clinical relevance. Mini Rev. Med. Chem. 6, 765–770 10.2174/138955706777698642 16842126

[28] Vitt U.A., Hsu S.Y. and Hsueh A.J. (2001) Evolution and classification of cystine knot-containing hormones and related extracellular signaling molecules. Mol. Endocrinol. 15, 681–694 10.1210/mend.15.5.0639 11328851

[29] Cheifetz S. (1987) The transforming growth factor-beta system, a complex pattern of cross-reactive ligands and receptors. Cell 48, 409–415 10.1016/0092-8674(87)90192-9 2879635

[30] Lee S.J. (2004) Regulation of muscle mass by myostatin. Annu. Rev. Cell Dev. Biol. 20, 61–86 10.1146/annurev.cellbio.20.012103.135836 15473835

[31] Guo R. (2016) Generation and evaluation of Myostatin knock-out rabbits and goats using CRISPR/Cas9 system. Sci. Rep. 6, 29855 10.1038/srep29855 27417210 PMC4945924

[32] Rauch F. and Schoenau E. (2001) The developing bone: slave or master of its cells and molecules? Pediatr. Res. 50, 309–314 10.1203/00006450-200109000-00003 11518815

[33] Rauch F and Schoenau E. (2000) Muscle, bone, and the Utah paradigm: a 1999 overview. Med. Sci. Sports Exerc. 911–917 10795780 10.1097/00005768-200005000-00006

[34] Frost H.M. (1997) On our age-related bone loss: insights from a new paradigm. J. Bone Miner. Res. 12, 1539–1546 10.1359/jbmr.1997.12.10.1539 9333113

[35] Bialek P. (2014) A myostatin and activin decoy receptor enhances bone formation in mice. Bone 60, 162–171 10.1016/j.bone.2013.12.002 24333131

[36] Byron C.D., Hamrick M.W. and Wingard C.J. (2006) Alterations of temporalis muscle contractile force and histological content from the myostatin and Mdx deficient mouse. Arch. Oral Biol. 51, 396–405 10.1016/j.archoralbio.2005.09.006 16263075

[37] Vecchione L. (2007) Craniofacial morphology in myostatin-deficient mice. J. Dent. Res. 86, 1068–1072 10.1177/154405910708601109 17959898

[38] Elkasrawy M.N. and Hamrick M.W. (2010) Myostatin (GDF-8) as a key factor linking muscle mass and skeletal form. J. Musculoskelet. Neuronal Interact. 10, 56–63 20190380 PMC3753581

[39] Jackson R.P., Peterson M.D., McManus A.C. and Hales C. (1998) Compensatory spinopelvic balance over the hip axis and better reliability in measuring lordosis to the pelvic radius on standing lateral radiographs of adult volunteers and patients. Spine 23, 1750–1767 10.1097/00007632-199808150-00008 9728376

