# Supplementary material for: ‘Double-muscling’ and pelvic tilt phenomena in rabbits with the cystine-knot motif deficiency of myostatin on exon 3
Source: Biosci Rep. 2019 May 21;39(5):BSR20190207. doi: 10.1042/BSR20190207 (PMC6527932; doi:10.1042/BSR20190207)
Supplement: Supplementary file 1 [file bsr20190207_Supp1.pdf]

Additional files

Table S1. Summary of heritability and breed of the MSTN-KO rabbits

F_0	F_1			F_2			F_2		
	$MSTN^{+/+}$	$MSTN^{+/a-}$	$MSTN^{+/b-}$	$MSTN^{+/+}$	$MSTN^{+/a-}$	$MSTN^{a-/a-}$	$MSTN^{+/+}$	$MSTN^{+/b-}$	$MSTN^{b-/b-}$
M2	4	3	1	10	10	2	0	0	0
M6	15	12	2	4	3	1	0	0	0
M8	2	6	7	4	3	6	6	7	0
M1	4	2	1	0	0	0	0	0	0
M3	4	1	1	0	0	0	0	0	0
M5	1	2	4	10	7	3	9	5	5
M10	15	0	0	-	-	-	-	-	-

Table S2. Hematology parameters of the *MSTN*$^{+/+}$, *MSTN*$^{+/-}$ and *MSTN*$^{-/-}$ rabbits.

**Parameter**	***MSTN*$^{+/+}$**	***MSTN*$^{+/-}$**	***MSTN*$^{-/-}$**
RBC(10^{12}/L)	5.35±0.60	5.67±0.65	4.77±0.34
HGB(g/L)	112.33±7.77	129.00±19.31	113.00±15.56
HCT(%)	34.73±2.18	38.73±5.15	32.10±2.12
MCV(fL)	68.63±1.98	68.33±1.27	70.35±3.89
MCH(pg)	22.30±1.61	22.63±0.85	23.60±1.56
MCHC(g/L)	335.33±5.51	332.00±5.57	335.50±3.54
WBC(10^9/L)	9.82±3.05	10.33±2.06	7.65±2.19
RDW－CV(%)	15.53±0.50	15.57±0.60	15.60±0.28
PLT(10^9/L)	340.67±65.65	280.00±123.77	392.50±142.13
MPV(fL)	6.00±0.46	6.00±0.10	6.00±0.42

Table S3. Biochemical parameters of the *MSTN*$^{+/+}$, *MSTN*$^{+/-}$ and *MSTN*$^{-/-}$ rabbits.

**Parameter**	***MSTN*$^{+/+}$**	***MSTN*$^{+/-}$**	***MSTN*$^{-/-}$**
ALT(U/L)	64.50±21.57	63.73±17.70	65.77±44.37
AST(U/L)	15.60±1.18	15.10±1.64	15.23±3.96
GLU(mmol/L)	6.27±0.57	6.70±0.20	5.93±0.47
TG(mmol/L)	0.34±0.07	0.28±0.02	0.37±0.13
TC(mmol/L)	1.37±0.52	1.43±0.50	1.68±0.54
CA(mmol/L)	2.72±0.12	2.82±0.17	2.70±0.11
P(mmol/L)	1.24±0.15	1.13±0.08	1.33±0.18
ALP(U/L)	17.33±2.08	20.67±5.86	17.00±2.65
LDH(U/L)	60.00±8.72	46.00±12.77	61.33±26.50

Table S4. The sequences of complementary DNA oligos.

Name	the pair of complementary DNA oligos
sgRNA-1	TATAgCCATGGTAGTAGACCGCTGT<br>AAACACAGCGGTCTACTACCATGGc
sgRNA-2	TATAgATCTTTGTGGGAGTACAGCA<br>AAACTGCTGTACTCCCACAAAGATc

Table S5. Primers for detection of *MSTN*-KO rabbits.

Name	5’-3’	TM
CAS9SG12-1	CTTATCGTTCTTTCCTTT	48.2℃
CAS9SG12-2	CCTATAGCCTATGGTACA	52.7℃

Table S6. The sequences of potential off-target sites.

A

	20	19	18	17	16	15	14	13	12	11	10	9	8	7	6	5	4	3	2	1	N	G	G
sgRNA-1	C	C	A	T	G	G	T	A	G	T	A	G	A	C	C	G	C	T	G	T	G	G	G
OT1	C	C	C	T	G	C	T	A	G	C	A	G	A	C	C	G	C	T	C	T	A	G	G
OT2	C	C	A	T	G	G	T	A	G	C	A	G	C	C	A	G	C	T	G	T	A	G	G
OT3	C	C	A	T	G	G	T	C	C	C	A	G	A	C	A	G	C	T	G	T	G	G	G
OT4	C	C	A	G	G	G	T	A	G	G	T	G	A	C	T	G	C	T	G	T	A	G	G
OT5	C	C	A	G	G	G	T	A	G	A	A	C	A	C	T	G	C	T	G	T	T	G	G

B

	20	19	18	17	16	15	14	13	12	11	10	9	8	7	6	5	4	3	2	1	N	G	G
sgRNA-2	A	T	C	T	T	T	G	T	G	G	G	A	G	T	A	C	A	G	C	A	A	G	G
OT6	G	T	G	T	T	T	G	A	G	G	G	A	G	T	A	C	A	G	C	A	A	G	G
OT7	A	G	T	T	T	G	G	T	G	G	G	A	G	T	A	C	A	G	C	A	A	G	G
OT8	A	C	C	T	A	T	T	A	G	G	G	A	G	T	A	C	A	G	C	A	A	G	G
OT9	A	T	C	T	T	G	G	T	G	G	G	G	G	T	A	C	A	G	C	A	G	G	G
OT10	C	T	C	T	T	T	C	T	G	A	G	A	G	T	A	C	A	G	C	A	G	G	G

A) OT1 to OT5 homologous to sgRNA-1. B) OT6 to OT10 homologous to sgRNA-2.

Table S7. The sequences of potential off-target loci PCR primers.

Primers	Primer sequences
OT-1	5′- TCCCTTCCTCGCTCCTAA -3′
	5′- TGTCCCTCTGTGCCTTGC -3′
OT-2	5′- TCCTGACTCCCGATTGC -3′
	5′- CCTGTGGCTCCTTATTGTT -3′
OT-3	5′- ACCCAAGGTGACTGTTGTTCC -3′
	5′- AAGGTCGCCATCGCCATTAC -3′
OT-4	5′- GGGCATCCTGGTTCCTCTT -3′
	5′- GGGCTCCCTGTGACTTGTG -3′
OT-5	5′- AACATACTCCCATCACTT -3′
	5′- GACTCCATTCAACCCT -3′
OT-6	5′- ATAGGCTTCTTTCTTGTTTC -3′
	5′- TCGTGCTGTAGCTGGTTGG -3′
OT-7	5′- GCCATCTTCTACTGCTATCC -3′
	5′- AATCCAACTCCCTACTAACG -3′
OT-8	5′- AATGGGTGGATGGATGGG -3′
	5′- TGGGAGGTAGTTGGAATA -3′
OT-9	5′- CCTCCCTCTGTTGTATTGC -3′
	5′- AAGCCAGTGGTGTCTGTTCT -3′
OT-10	5′- TTGCCACAAATACATCGG -3′
	5′- CCAGGGCTTCATACTACTCA -3′
